# Intestinal flora plays a role in the progression of hepatitis-cirrhosis-liver cancer

**DOI:** 10.3389/fcimb.2023.1140126

**Published:** 2023-03-09

**Authors:** Shuyu Liu, Xilan Yang

**Affiliations:** Department of General Practice, The Fourth Affiliated Hospital of Nanjing Medical University, Nanjing, China

**Keywords:** intestinal flora, bacterial translocation, dysbiosis, hepatitis, cirrhosis, liver cancer

## Abstract

The liver is a vital metabolism and detoxification organ of human body, which is involved in the biotransformation and metabolism of the organism. Hepatitis - cirrhosis - liver cancer are significant and common part of liver diseases. The pathogenesis of liver diseases is generally as followed: inflammation and other pathogenic factors cause persistent damage to the liver, leading to the activation of hepatic stellate cells (HSCs) and excessive deposition of extracellular matrix. Patients with chronic hepatitis have a high risk of developing into liver fibrosis, cirrhosis, and even life-threatening liver cancer, which poses a great threat to public health.As the first organ to come into contact with blood from the gut, the liver is profoundly affected by the intestinal flora and its metabolites, with leaky gut and flora imbalance being the triggers of the liver’s pathological response. So far, no one has reviewed the role of intestinal flora in this process from the perspective of the progression of hepatitis-cirrhosis-liver cancer and this article reviews the evidence supporting the effect of intestinal flora in the progression of liver disease.

## 1 Introduction

The causes of hepatitis are diverse: alcohol, virus, drug-related, etc. According to the World Health Organization, at least 296 million people worldwide have chronic hepatitis B virus (HBV) infection, and the number of deaths caused by cirrhosis and hepatocellular carcinoma due to HBV infection is approximately 820,000 (Sung et al., 2021). The persistent infection of hepatitis B virus is a worldwide public health problem, so this paper focuses on hepatitis B. Chronic hepatitis B (CHB) renders sustaining liver damage, resulting in cirrhosis and even liver cancer as the disease progresses, but not everyone progresses to liver cancer and there are individual differences, suggesting that there are a number of genetic and environmental influences in addition to the disease itself. The intestinal flora has recently been recognised as a major environmental factor influencing the pathogenesis of several human diseases, including cirrhosis and liver cancer (Tilg et al., 2016; Yang et al., 2020).

The intestinal flora is a complex and dynamic community composed of bacteria, fungi, protozoa, vibrios and viruses. Current research mainly focuses on bacteria among these microbiota.There are nine main phyla of intestinal bacteria, of which Firmicutes and Bacteroidetes are the dominant ones. Under normal circumstances, intestinal flora maintains a certain balance, but when the intestinal barrier function changes and the intestinal environment changes,some conditional pathogenic bacteria such as Escherichia coli and Proteus multiply in the intestine, resulting in intestinal flora imbalance and causing body diseases.The liver and intestine communicate bilaterally through the portal vein, bile duct and systemic circulation. The interaction between gut microbiota and gut-liver forms the gut-liver axis. Based on the gut-liver axis, intestinal flora regulates pro-inflammatory changes in the liver and intestine, thereby affecting the development of hepatitis, liver fibrosis, cirrhosis and hepatocellular carcinogenesis (Woodhouse et al., 2018).

A large number of clinical and experimental evidence supports the role of intestinal flora in various liver diseases. However, its role in the development and progression of hepatitis-cirrhosis-hepatocellular carcinogenesis has not been outlined.This article focuses on a review of the laboratory evidence supporting this link.

## 2 Gut–liver axis and the intestinal barrier

**Figure 1:**
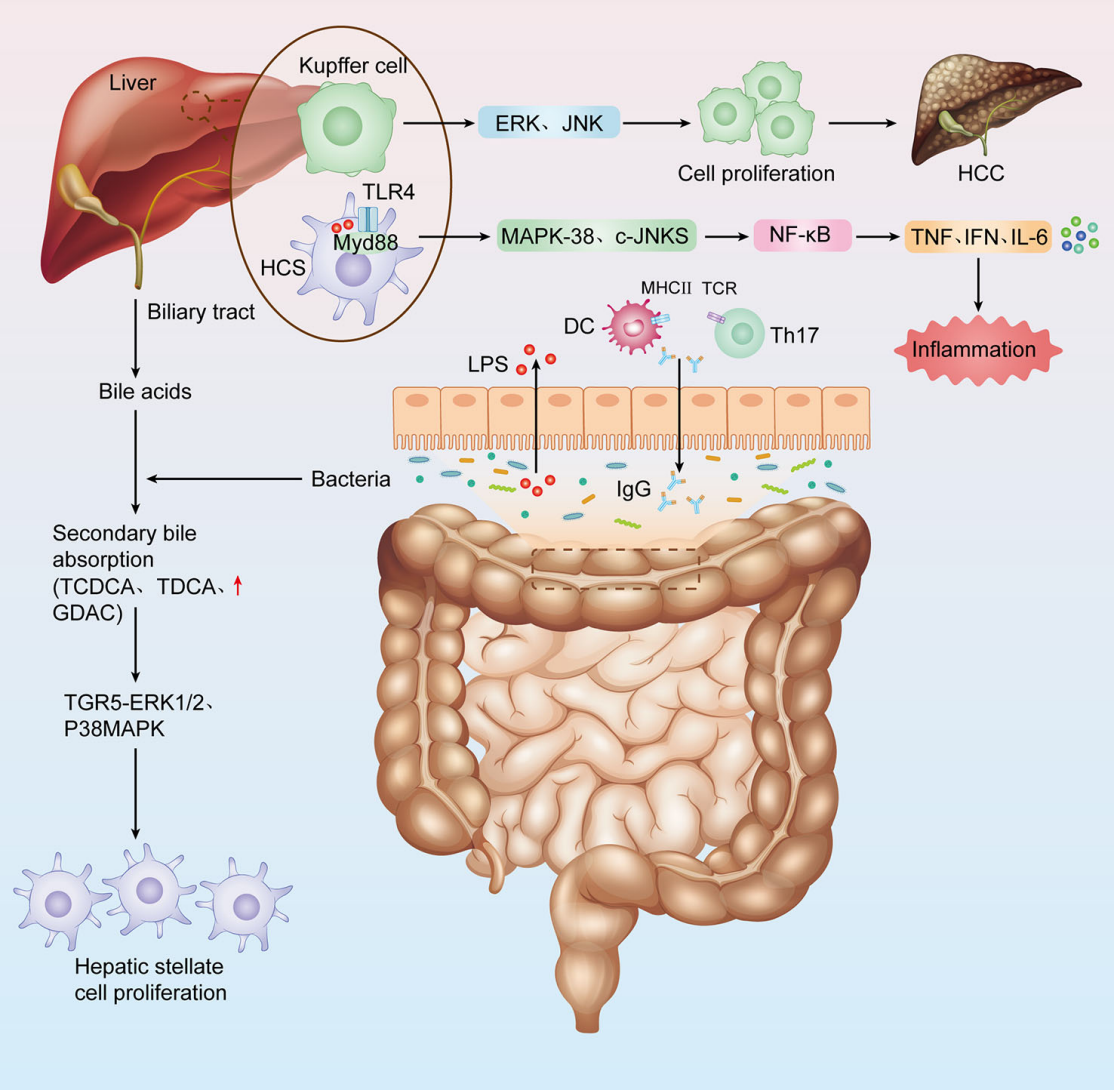
The link between intestinal flora and hepatitis-cirrhosis- Hepatocellular carcinoma. Impaired liver function, decreased bile secretion and albumin synthesis, and portal hypertension all cause decreased intestinal mucosal barrier function, intestinal flora imbalance, increased bacterial load, bacterial translocation, and bacteria and their metabolites aggravate liver injury.

The gut-liver axis is a bidirectional communication pathway composed of intestinal flora, hepatic portal system and biliary system, which is the physiological basis for the interaction between intestinal flora and liver (Yang et al., 2020). The liver is the first organ exposed to blood from the intestine, which accounts for about 70% of the liver blood supply. Under physiological conditions, the liver transmits nutrients to the intestine. The intestinal mucosal barrier ensures the intestinal absorption of nutrients while limiting the absorption of pathogenic bacteria and microbiota-derived molecules ((lipopolysaccharide (LPS), bacterial DNA, flagellin, peptidoglycan, etc.))into the portal circulation and liver. The intestinal mucosal barrier is an anatomically functional structure consisting of (i) a mechanical barrier: intact intestinal mucosal epithelial cells and tight intercellular junctions, (ii) an immune barrier: intestinal-associated lymphoid tissue including: intraepithelial lymphocytes, lymphoid follicles, Peyer’s cells and mesenteric lymph nodes, and (iii) a secretory barrier: mucus secreted by intestinal mucosal cup cells, immunoglobulin (Ig) A and antimicrobial products. These three components make up the intestinal mucosal barrier (Sanduzzi Zamparelli et al., 2017; Guo et al., 2022; Li et al., 2022). However, in a state of liver injury, the intestinal mucosal barrier function is disrupted and the intestinal flora and its metabolites can reach the liver as antigenic signals, inducing inflammatory responses and immune regulation, while the liver influences the number and composition of the intestinal flora by regulating the secretion of bile acids and the signalling pathways mediated by them (Wei et al., 2013; Iannacone and Guidotti, 2022). Intestine flora promotes the progression of liver diseases by regulating proinflammatory/immune signaling pathways (Albhaisi et al., 2020) (**Figure 1**).

## 3 Intestinal flora and chronic hepatitis B

Simple HBV infection does not directly cause liver injury,and immune-mediated hepatocellular injury is the main cause of liver injury in chronic viral hepatitis B.The cytotoxic immune response of the host against virus-infected hepatocytes can lead to liver injury, for example, cytotoxic lymphocytes (e.g. natural killer cells) or virus-specific cytotoxic T lymphocytes (CTL) eliminate infected hepatocytes, cytokines secreted by activated lymphocytes and monocytes, and cause hepatocyte destruction while eliminating virus from hepatocytes (Iannacone and Guidotti, 2022). Natural killer cells belong to the innate immune system, but they do not express conventional T-lymphocyte receptors.Instead, they express receptors that recognize the major histocompatibility complex (MHC) that is abnormally expressed by virus-infected cells. These virus-infected cells are destroyed by exocytotic particles(containing apoptosis initiation factors) released by natural killer cells (Bertoletti and Naoumov, 2003; Peter et al., 2015). The CTL is stimulated by a dual signal of the MHC/antigenic peptide/T-lymphocyte receptor complex as the first signal and the combination of CD95 on the target cell and CD95L on the CTL as the second signal, which releases a large number of cytokines, perforins, granuprotease, etc. The perforins released by the CTL can form pores in the hepatocyte membrane, which allow cytokines and granuprotease to enter the cell. CD95/CD95L binding activates a death-inducing signalling complex consisting of many molecules, including Procaspase 8/10, which activates caspase-8 and activates the cascade of caspases,and also induces apoptosis (Wang et al., 2022). In one study, mice treated with vancomycin (Van) and gentamicin (Gen), respectively, were found to have a reduced CD95/CD95L-mediated hepatocyte apoptosis in Gen-treated mice by inhibiting CD95 expression on the surface of hepatocytes. Further using 16sRNA sequencing and correlation analysis, we isolated the five strains with the highest relative abundance from the feces of Gen-treated mice, of which only *Bacteroides acidifaciens* was protective against liver injury, which may become a new target for the treatment of liver diseases in the future (Kassa et al., 2021; Wang et al., 2022). Recent studies have found that the natural immune response induced by pathogen-associated molecular patterns(PAMPs) generated by dysbiosis of the intestinal flora also plays an important role in the progression of hepatitis B. Pathogen-associated molecular patterns (PAMPs) are a type of pattern recognition receptors (PRRs), and Toll-like receptors (TLRs) are one of the important members of PRRs. PAMPs are recognized by human immune cells by binding to TLRs and induce immune responses(West and Heagy, 2002; Jiao et al., 2020). TLR4 is expressed by a variety of cells in the liver, such as Kupffer cells and hepatic stellate cells (HSC). Dysbiosis of the intestina flora increases endotoxin (LPS) load. LPS binds to LPS binding proteins, and TLR4 recognizes this binding, inducing signaling cascades (e.g., the nuclear factor-κb pathway) and secreting cytokines (e.g., IL1, IL-6, TNF-α), which trigger chronic inflammation. Due to the special anatomical location between the liver and the intestine, which requires frequent exposure to LPS from intestinal sources, and long-term exposure to LPS at low levels,chronic low-level exposure to LPS without signs of inflammation in the healthy liver is a phenomenon known as endotoxin tolerance, on the one hand, because of the relatively low expression of TLR4 and its receptor molecules in the liver, on the other hand, in the case of a healthy organism, the liver negatively regulates Toll-like receptor 4 signaling pathway at different levels (Luedde and Schwabe, 2011). If TLR4 is exposed to increased lipopolysaccharide(LPS) expression, as well as increased TOLL-like receptor 4 sensitivity, disruption of hepatic tolerance may result in an inappropriate immune response, leading to chronic inflammatory liver disease. Other translocated intestinal bacteria can also activate inflammatory responses throughTLRs, and TLR2 is involved in the recognition of the cell wall components peptidoglycan (PGN) and lipophosphate (TA) in Gram-positive bacteria (Akira et al., 2006; Kawai and Akira, 2010). TLR3 recognizes double-stranded RNA (dsRNA) in bacteria,TLR5 recognizes bacterial flagellin and TLR-9 recognizes unmethylated CpG DNA,and TLR7 expressed on conventional dendritic cells (cDC) senses RNA species from bacteria, such as group B Streptococcus,and induces type I interferon (Mancuso et al., 2009; Coban et al., 2010; Kawai and Akira, 2010; Guiducci et al., 2013). The final downstream effect following recognition of the above signals is to induce the production of inflammatory cytokines and type I interferons *via* NF-κB or IRF7, respectively(Shen et al., 2023). On the other hand, patients with hepatitis have impaired liver function, reduced bile secretion and albumin synthesis, causing edema in the intestinal wall and affecting intestinal blood supply and peristalsis, resulting in intestinal mucosal barrier damage and increased permeability. Impaired intestinal mucosal barrier function can lead to the translocation of bacterial components such as lipopolysaccharide, bacterial DNA, peptidoglycan, extracellular vesicles and their metabolites into the liver, and alteration in the composition and function of intestinal flora (reduced number of beneficial bacteria, reduced colonisation resistance,excessive growth of pathogenic bacteria, etc.), which aggravates liver injury by activating immune cells and inflammatory cells.

In recent years, many scholars have explored the diversity of intestinal flora and the role of its products in hepatitis B (Lu et al., 2011; Zeng et al., 2020; Li et al., 2022). The microbiome of patients with chronic hepatitis B exhibits ecological dysbiosis. Zeng et al. (2020) et al. found in their study that compared with healthy people,the abundance of Firmicutes was lower and that of Bacteroidetes was higher at the phylum level in CHB patients,the potential mechanism may be that Bacteroidetes are dominated by Gram-negative bacteria, which are the main donors for the synthesis of endotoxin LPS,and more endotoxin load promotes the progression of chronic liver disease,while Firmicutes were dominated by Gram-positive bacteria. At the family level, Enterobacteriaceae, a taxon containing a variety of pathogenic bacteria (such as Klebsiella, Escherichia coli, Proteus, Enterobacteriaceae, and other potential pathogenic bacteria), is highly abundant.This may be related to the fact that Enterobacteriaceae are mostly ethanol-producing bacteria, which can regulate autophagy through the IL-17 signaling pathway to induce liver injury (Yuan et al., 2019; Li et al., 2021). Bifidobacteriaceae and Clostridiaceae, two groups composed of many beneficial bacteria, were significantly decreased. Bifidobacteriaceae is one of the most important probiotics, which can benefit human health by reducing plasma and intestinal endotoxin levels, regulating intestinal flora composition, producing antimicrobial factors, improving intestinal barrier function, and regulating local and systemic immunity (Xu et al., 2012; Wan and El-Nezami, 2018). There shows a decrease in Clostridium spp. and Rumenococcus spp. in CHB patients,which resulted in a decrease in the production of short-chain fatty acids (SCFAs).Short-chain fatty acids are a variety of indigestible polysaccharides that are fermented by intestinal bacteria to become absorbable in the intestine, including propionate, butyrate and acetate (Huang et al., 2018; Wang et al., 2018). SCFAs can activate G protein-coupled receptors to trigger intracellular signaling or act as histone deacetylase inhibitors to regulate gene expression (Yu and Zhong, 2022). In addition, SCFAs can also participate in intestinal immunity by regulating the differentiation and activation of Treg cells or proinflammatory TH1 and TH17 cells, plasma cell differentiation and IgA secretion (Zhang et al., 2019). Butyric acid is the most direct source of energy for intestinal mucosal cells and plays an important role in maintaining the homeostasis of the intestinal mucosa (Thibault et al., 2010; Canani et al., 2011; Donohoe et al., 2012). In a study conducted by Pant et al. (2019), it was found that HepG2.2.15 cells (HBV-expressing cells) of CHB patients with a high viral load had higher expression of histone deacetylase (SIRT-1) and lower Ac-p53 levels than CHB patients with a low viral load. Butyrate can increase the expression level of Ac-p53 by inhibiting SIRT-1, and the up-regulated Ac-p53 can inhibit the expression of p-akt and cyclin D1, thus inhibiting cell proliferation, thus we speculate that butyrate may inhibit HBV replication and host cell proliferation through SIRT-1.Interestingly, another study showed that SCFAs (especially butyrate) produced by inulin digestion promote the development of hepatocellular carcinoma (HCC), and inulin induces HCC through early cholestasis, hepatocyte death, and subsequent development of hepatic neutrophilic inflammation (Singh et al., 2018).

## 4 Intestinal flora and chronic hepatitis C and E

Compared with CHB, the intestinal flora of patients with Chronic hepatitis C(CHC) and Chronic hepatitis E(CHE) has been less studied. HCV infect 130-210 million people worldwide, and HCV is another important risk factor for end-stage liver disease such as cirrhosis and HCC (Pradat et al., 2018). HCV infection is easy to become chronic, about 50-80% of patients become chronic infection, which may be related to the high variability of HCV, its pantropism towards extrahepatic cells and the low titer and weak immunogenicity of HCV in the blood. HCV enters the body and first causes viremia, which appears intermittently throughout the course of the disease. HCV RNA can be detected in blood or liver tissue within the first week. Endotoxemia is also common in patients with chronic hepatitis C, and there is a significant positive correlation between the severity of endotoxemia and viral replication as well as laboratory tests (alanine and aspartate aminotransferase levels, and lobular necrosis) (Sozinov, 2002). Dolganiuc et al. (2007). Found that LPS, IFN-γ, and HCV core protein act synergistically to induce and maintain monocyte/macrophage activation, leading to the loss of the body’s tolerance to TLR and ultimately leading to the persistent inflammatory state in HCV patients. LPS levels are closely related to the persistent inflammatory response in patients with viral hepatitis. On the other hand, HCV can induce a large number of dysfunctional CD4+T cells and CD8+T cells. Immune activation in the host as it clears HCV may lead to chronic inflammation. It is the persistent chronic inflammatory state that leads to the development of progressive fibrosis and cirrhosis (Rehermann and Thimme, 2019; Wang et al., 2022). HCV causes liver damage through direct killing, host immunity (CD4+ T cells and CD8+ T cells), autoimmunity, apoptosis and other mechanisms, with consequent reduction in bile salt secretion and protein synthesis, ultimately leading to bacterial overgrowth and changes in the intestinal microenvironment and microbial community composition (Duboc et al., 2013; Ridlon et al., 2014; Aly et al., 2016). HCV can also play a role in the homeostasis of the intestinal flora by regulating the secretion of bile acids. Heidrich et al. (2018) and Inoue et al. (2018)‘s study found that intestinal flora changes in CHC patients were mainly characterized by an increase in Enterobacteriaceae and Bacteroidetes, but a decrease in Firmicutes. Some pathogenic bacteria, such as Enterobacteriaceae, Staphylococcus, and Enterococci, reduced bile acid levels in HCV-infected patients with cirrhosis. Antiviral treatment with ribavirin (RBV) and the immunomodulator pegylated interferon (PEG-IFN) for HCV has no direct effect on intestinal dysbiosis, but the treatment increases bile acid, which is important for regulating intestinal flora. (Ponziani et al., 2018). Aly et al. (2016) and Preveden et al. (2017)‘s studies shows that HCV patients were more enriched in Prevotella spp. and Faecaliberium spp. while the healthy subjects were more enriched in Ruminococcus spp., Bifidobacterium spp. and Clostridium spp. Possible reasons behind this change in microbial composition: (i) Prevotella plays a role in promoting carbohydrate fermentation, and in HCV patients, impaired digestion and absorption may lead to increased carbohydrate concentrations in the colon. In addition, Prevotella also contains enzymes that play an important role in mucin degradation, which may lead to increased intestinal permeability. (ii) Due to the common embryonic origin of hepatocytes and gastric cells, HCV can also invade gastric cells and infect gastric B lymphocytes, leading to the decrease of IgA secretion (Aly et al., 2016). IgA can regulate the composition and abundance of intestinal microbiome, so reduced IgA secretion leads to increased abundance of Prevotella and other intestinal flora structure and function disorders. In addition, reduced IgA secretion increases intestinal permeability, leading to increased bacterial translocation (Mantis et al., 2011; Milosevic et al., 2021). It has also been suggested that Lactobacillus acidophilus and Bifidobacterium can be used as a complementary treatment to effectively reduce LPS in patients with hepatitis C. Lactitol is known as a prebiotic, and Chen et al. (2007) et al. found that oral administration of lactitol not only modulates gut microbiota, but also reduces plasma LPS in HCV patients more effectively than conventional treatment. It can be used as an effective adjuvant method for the treatment of HCV. A study by Pérez-Matute et al. (2019) found that alpha diversity was not recovered after clearance of hepatitis C virus from the body with novel direct antiviral agents (DAAs) in patients with HCV infection but not yet cirrhosis. However, after 3 months of observation, they found partial recovery of inflammation, alpha diversity in patients with less fibrosis and therefore early initiation of DAA therapy may have a role in improving the dysbiosis of the intestinal flora caused by HCV infection.

The pathogenesis of hepatitis E is not well known. Acute viral hepatitis caused by hepatitis E virus infection is an important community health problem, and hepatitis E is a self-limiting disease with no direct pathogenic effect on hepatocytes. As hepatitis E is transmitted by the faecal-oral route, it may have a devastating effect on the intestinal flora (Lemon et al., 2017). Wu et al. (2022)‘s study found that acute hepatitis E(AHE) was more enriched in Proteobacteria, Gammaproteobacteria, and Enterobacteriaceae compared to healthy controls (HCs). The abundance of Gammaproteobacteria can distinguish AHE patients from HCs and play a role in predicting the severity of AHE patients.In a study by Kreuzer et al. (2012), two groups of sows and piglets were fed a conventional diet and a diet supplemented with healthy probiotics such as Enterococcus faecalis NCIMB 10415, and it was found that the probiotics affected the reduction and clearance of intestinal HEV virus in pigs, which may be related to immune factors, but the exact mechanism remains to be elucidated. Kuss et al. (2011) found in a study that enteroviruses use intestinal microbes to enhance replication and transmission and that antibiotic-mediated microbiota depletion is effective in reducing viral infections.In another study, it was found that reducing the number of bacteria in the host animal significantly reduced the number of hatching T. muris eggs, and thus, intestinal microbes could induce the hatching of intestinal nematodes in mice (Hayes et al., 2010). The above study shows that a variety of pathogens use bacteria (microbiota) to proliferate, so can it be speculated that intestine microbes also play an important role in the proliferation of hepatitis viruses? More research is needed to identify and validate the specific mechanisms by which hepatitis viruses utilize intestine microbes.

## 5 Intestinal flora and liver cirrhosis

There are two main features of liver cirrhosis: liver structural disorder and portal hypertension, which are mutually causal and promote the progression of the disease. On the one hand, portal hypertension, causing structural changes in the intestinal wall, including vascular congestion, oedema, fibromuscular hyperplasia, thickening of the mucosal muscle layer and reduction or loosening of the tight junctions (TJ), and an increase in the intestinal flora and endotoxin load, leads to the activation of PRR on immune cells, especially macrophages, resulting in the activation of quiescent HSC into activated HSC (Hrncir et al., 2021; Kassa et al., 2021). Subsequently, activated HSC proliferate in response to various cytokines, secreting type I collagen fibres and forming liver fibrosis (Kendall et al., 2009). Moreover, TLR4 is expressed in hepatic stellate cells (HSC), where increased LPS load releases large amounts of LPS-TLR4 pathway-dependent extracellular matrix proteins that promote the progression of liver fibrosis (Kassa et al., 2021), On the other hand, by activating TLR-4, it initiates intrahepatocellular signaling cascades, causing activation of nuclear factor NF-κβ, P38 MAPK and JNK, triggering immune inflammation and sustained inflammatory stimulation, resulting in regenerative repair of hepatocytes after repeated damage and promoting the progression of liver cirrhosis (Zheng et al., 2020). Studies have found that reduced gastric acid secretion and altered bile acid secretion in patients with cirrhosis resulting in impaired intestinal motility, reduced antimicrobial activity of defensins, and reduced mucosal IgA level. Bile acids exert antibacterial effects by directly destroying bacterial cell membranes and indirectly acting as signaling molecules by binding and activating farnesol X receptor (FXR), the nuclear receptor for bile acids (Wiest et al., 2014; Ridlon et al., 2015). FXR also regulates the expression of genes that play a crucial role in preventing bacterial overgrowth and maintaining intestinal epithelial integrity (Inagaki et al., 2006). Interestingly, dysbiosis of intestinal flora alters the levels of bile acid metabolites, with most conjugated bile acids,such as TCDCA, TDCA, and GDCA increasing and TDCA or GDCA promoting the proliferation of hepatic stellate cells and the formation of liver fibrosis by activating the TGR5-ERK1/2 and P38 MAPK signaling pathways (Inagaki et al., 2006). In summary, intestinal flora promotes the development and progression of cirrhosis and its complications by disrupting the mucosal barrier or translocation of intestinal flora and its products.

Qin et al. (2014)showed that Bacteroidetes and Firmicutes dominated the intestinal flora in the feces of the healthy control group and the cirrhosis group at the phylum level.Compared with healthy controls, patients with cirrhosis had fewer Bacteroidetes but more Proteobacteria and Fusobacteria.At the genus level,*Bacteroides* was the dominant species in both groups,with a significant decrease in the cirrhosis group. Among the remaining genus, *Veillonella, Streptococcus, Clostridium* and *Prevotella* were enriched in the cirrhosis group, and *Eubacterium* and *Alistipes* were enriched in the healthy control group. Among the 20 species with the highest number increase in the cirrhosis group, four were *Streptococcus* spp. and six were *Clostridium* spp. suggesting that these two genus may play an important role in cirrhosis. Of the species with the greatest decrease in number in the cirrhosis group, twelve were Bacteroidetes and seven were Firmicutes, especially from the Clostridium. Interestingly, Enterobacteriaceae and Streptococcaceae were enriched in patients with cirrhosis and positively correlated with Child-Pugh scores, and another study also showed similar results. The microbiota of patients with compensated cirrhosis (Child-Pugh A and B) and decompensated cirrhosis (Child-Pugh C) were significantly different,with significantly higher levels of Proteobacteria, particularly Enterobacteriaceae, in patients with decompensated cirrhosis (Liu et al., 2018). This may be due to the fact that bacteria of Enterobacteriaceae (including *Escherichia coli, Klebsiella, Proteus* and *Enterobacter*) are all considered to have PAMPs (Chen et al., 2011; Lu et al., 2011; Bajaj et al., 2012a), among which *Proteus mirabilis* and *Escherichia coli* can also activate the NLRP3 inflammasome to promote monocytes to release a large amount of IL-1β and aggravate inflammatory damage (Hirota et al., 2011; Seo et al., 2015; Liao et al., 2019). In terms of functional metabolism, compared with the healthy group, the enrichment of ammonia-producing modules in the cirrhosis group indicates that the intestinal flora produces excessive ammonia, and the GABA biosynthesis module is enriched in the patients, and that the GABA neurotransmitter system is involved in the pathogenesis of human hepatic encephalopathy. Bajaj et al. (2012b) also reached a similar conclusion, they found that specific bacterial families such as Alcaligeneceae, Porphyromonadaceae, Enterobacteriaceae were strongly associated with cognition and inflammation in patients with hepatic encephalopathy. All these findings suggest that dysbiosis of the intestinal flora plays an important role in hepatic encephalopathy, a complication of cirrhosis. With the progress of research, many clinical studies have found that dysbiosis of the intestinal flora also plays an important role in other complications of liver cirrhosis, including ascites formation and infection. *Escherichia coli* and *Streptococcus* are significantly enriched in patients with co-infections in cirrhosis (Riordan and Williams, 2006; Chen et al., 2011; Kakiyama et al., 2013).

## 6 Intestinal flora and hepatocellular carcinoma

The pathogenesis of HCC is diverse and HCC is often characterised by a high level of immune cell infiltration. Therefore, immune status may play a significant role in the progression of HCC. Helper T lymphocytes (Th) 17 are capable of promoting tumour angiogenesis through the production of pro-inflammatory and pro-angiogenic mediators, such as IL17, IL-8 and TNF-a (Kryczek et al., 2009; Su et al., 2010), while the tumour microenvironment also mediates Th17 cell chemotaxis and accumulation at the tumour site (Miyahara et al., 2008; Zou and Restifo, 2010; Ye et al., 2013). IL-17 may enhance the proliferation of hepatocellular carcinoma cells by activating the IL-6/STAT3 pathway (Hu et al., 2017). Th17 levels in the liver of healthy individuals are low and help protect the intestinal mucosa from pathogens, and its differentiation depends on the cellular response to commensal bacteria, and studies have shown that intestinal segmented filamentous bacteria can induce differentiation of Th17 cells by activating intrinsic dendritic cells in the intestinal mucosa (Hu et al., 2017). The flagellin on the surface of Salmonella upregulates the expression of major histocompatibility complex II(MHCII) molecules, CD80 and CD86, by binding to and activating TLR5 on the surface of dendritic cells in the lamina propria of the intestine, and secretes interleukin-23 (IL-23), which promotes the activation of B lymphocytes to secrete immunoglobulins (Cunningham et al., 2004; Sanders et al., 2006; Bates et al., 2009). On the other hand, flagellin as the only ligand of TLR5, stimulates innate immunity. Studies have found a novel bacterial flagellin:CBir1 produced by Clostridium, is recognized by CBir1 T cells and induces TH17 differentiation in the intestine (Liu et al., 2016). In addition, there is an interaction between intestinal flora and programmed death ligand-1 (PD-L1) and cytotoxic T-lymphocyte antigen-4 (CTLA-4). The key role of *Bifidobacterium* and *Bacteroides* in blocking the immunostimulatory effects of PD-L1 and CTLA-4, respectively, was identified through the establishment of a mouse tumour model (Sivan et al., 2015; Vétizou et al., 2015). The intestinal flora also affects the immune system through various metabolites that are released into the circulation through the intestinal barrier. For example, SCFAs play important roles in the differentiation and activation of regulatory T cells (Treg cells) or Th17 and 1 cells and immunoglobulin A (IgA) -producing B cells (Zhang et al., 2019; Lee et al., 2020; Yu and Zhong, 2022). Intestinal flora can also signal to immune cells in gut-associated lymphoid tissue and mesenteric lymph nodes by influencing the family of pattern recognition receptors (PRRs) to promote immune responses (Mogensen, 2009; Bermejo-Jambrina et al., 2018). For example, high levels of LPS in the liver activate TOLL-like receptor 4 (TLR4) in Kupffer cells and hepatic stellate cells (HSC), and the LPS-TLR4 pathway is a key initiator of HCC development, acting mainly through: (i) After excessive LPS into the liver, it is recognized by TLR4 on Kupffer cells, and the LPS-TLR4 signal transduction pathway is activated, leading to increased production of inflammatory mediators, promoting liver inflammation and oxidative damage, inhibiting cell apoptosis, and causing DNA damage(Dapito et al., 2012), (ii) Studies have shown that LPS-TLR4-induced activation of ERK and JNK signaling promotes cell proliferation by regulating Bax translocation to mitochondria, which plays a role in promoting the survival and proliferation of hepatocellular carcinoma cells (Wang et al., 2013; Yang et al., 2015), (iii) LPS activates myeloid differentiation protein 88 (MyD88) -dependent LPS-TLR4 signaling pathway by recognizing TLR4 receptor on hepatic endothelial cells (LECs). MyD88 regulates angiogenesis by regulating extracellular proteases, and angiogenesis is a key link in the progression of HCC,allowing cirrhosis to progress to HCC (Jagavelu et al., 2010). LPS can also induce hepatic progenitor cells (HPCs) to differentiate into myofibroblasts. By secreting IL-6 and TNF-α, it further induces the activation of tumor-related signaling pathway Ras and the inactivation of tumor suppressor signaling pathway p53 in HPCs, thereby ultimately promoting the abnormal proliferation and transformation of HPCs and hepatocarcinogenesis (Ren et al., 2019; Gram and Kowalewski, 2022). Gut microbes also control the aggregation of hepatic natural killer T (NKT) cells through the BA-CXCL16-CXCR6 axis, and NKT cells initiate hepatic anti-tumour immune mechanisms through the production of IFN-γ (Zhang et al., 2019). CXCL16 is the only ligand for the chemokine receptor CXCR6, which mediates the accumulation of NKT cells (Garrett, 2019), and primary bile acids increase CXCL16 expression, whereas secondary bile acids show the opposite effect (Ponziani et al., 2018). Whereas intestinal flora mediates the metabolism of bile acids (BA) in the gut, when intestinal flora is dysregulated, hepatic bile acid metabolic homeostasis is disrupted, toxicity levels increase and liver tumour progression is promoted *via* the BA-CXCL16-CXCR6 axis (Ponziani et al., 2018). In addition, certain bacterial species can be indirect causes of disease by stimulating an inflammatory state, such as inflammation by Enterotoxigenic Bacteroides fragilis (ETBF) induces cancer development through Stat3 and Th17-dependent pathways (Wu et al., 2009; Boleij et al., 2015), while an inflammatory state also increases the production of reactive oxygen species (ROS) which induces DNA damage and promotes tumour progression, e.g. Enterococcus faecalis induces ROS production in colonic epithelial cells to induce DNA damage (Mangerich et al., 2012; Kostic et al., 2013; Sun et al., 2017). Dapito et al. (2012) treated mice with low-dose non-toxic LPS *via* a subcutaneous osmotic pump for 12 weeks in a mouse model of diethylnitrosamine (DEN) and hepatotoxin carbon tetrachloride (CCl4) induced hepatocellular carcinoma. This low-dose LPS treatment resulted in significant increases in inflammatory gene expression, tumor number, tumor size, and liver weight to body weight ratio, further confirming that our LPS-TLR4 pathway promotes HCC development, including mediating cell proliferation, promoting hepatocyte mitogenic epiregulin expression, and inhibiting apoptosis, suggesting that intestinal flora and TLR4 may be therapeutic targets to prevent liver cancer progression. In another HCC mouse model, the beneficial effects of probiotics on liver immune differentiation and carcinogenesis were confirmed by using a novel probiotic mixture (Prohep). Prohep treatment slowed tumor growth and reduced tumor volume by 40% compared with untreated mice.The levels of Prevotella and Oscilibacter were increased in the fecal microbiota of mice treated with probiotics, indicating that the anti-inflammatory effect of these beneficial bacteria can positively affect hepatocarcinogenesis (Li et al., 2016). We have listed the composition of the intestinal flora in the studies mentioned above on patients with hepatitis, cirrhosis and hepatocellular carcinoma in **Table 1**.

**Table 1:** Intestinal flora composition in patients with hepatitis, cirrhosis and HCC.

Author/Date	Population	Number	methodology	PHYLUM	CLASS	ORDER	FAIMLY	GENUS
Shen et al. (2023)	HBV-CLD Health control	64 17	16s rRNA					↑Fecalibacterium, ↑Streptococcus, ↑Sutterella, ↑Lachnospiraceae ↑Blautia, ↑Escherichia, ↑Shigella, ↑Klebsiella, ↑Collinsella, ↑Lactococcus
Zheng et al. (2020)	Hepatitis liver cirrhosis HCC Health control	24 24 75 20	16s rRNA	↑Verrucomicrobia↑Proteobacteria ↓Tenericutes ↑Fusobacteria ↑Verrucomicrobia ↑Proteobacteria ↓Tenericutes				↑Phyllobacterium, ↑Sphingomonas, ↑Enterococcus, ↑Romboutsia ↓Ralstonia, ↓Catenibacterium, ↓Lachnospira ↑Mitsuokella, ↓Ralstonia
Li and Kang (2022)	CHB-immune-tolerant (IT) CHB -immune-active (IA) Control	14 10 13	16s rRNA	↑Bacteroidetes ↑Firmicutes, Actinobacteria↓ ↑Firmicutes, ↑Actinobacteria ↑Actinobacteria				↑Bacteroides, ↑Prevotella, ↑Megamonas ↑Blautia ↑Faecalibacterium ↓Bacteroidetes ↑Eubacterium rectale ↑Eubacteriumhallii , ↑Bifidobacterium,
Chen et al. (2011)	Liver cirrhosis Health control	36 24	16s rRNA , real-time quantitative polymerase chain reaction.	↑ Proteobacteria ↑ Fusobacteria ↓Bacteroidetes			↑Enterobacteriaceae ↑ Veillonellaceae ↑ Streptococcaceae	↓Lachnospiraceae
Yang and F. Lv, R.(2020)	HBV carrier Chronic Hepatitis B Cirrosis Acute-on-chronic liver failure Health control	24 56 54 52 31	16s rRNA gene sequencing					↑Prevotella ↑Lachnospira ↑Ruminococcus ↑Clostridium ↑Bacteroides ↑Veillonella ↑Erysipelatoclostridium ↑Veillonella ↑Erysipelatoclostridium ↑Enterococcus ↑Lactobacillus ↑Veillonella ↑Erysipelatoclostridium ↑Roseburia ↑Faecalibacterium
Kakiyama et al. (2013)	Liver cirrhosis(childB/C) Liver cirrhosis(childA ) Control	24 23 14	culture-independent multitagged-pyrosequencing				↑Enterobacteriaceae ↑Veillonellaceae (a) ↓Lachonospiraceae ↓Ruminococcaceae ↓ Blautia (a)	
Wei et al. (2013)	hepatitis B liver cirrhosis (HBLC). health control	20 20	16S rRNA gene high-throughput Solexa sequencing Real-time qPCR analysis	↓Bacteroidetes ↑Proteobacteria	↑Negativicutes ↑Bacilli	↑Enterobacteriales ↑Selenomonadales ↑Lactobacillales	↑Enterobacteriaceae ↑Veillonellaceae ↑Streptococcaceae	
Ren and Li (2017)	Hepatocellular carcinoma(HCC) Cirrhosis Health control	75 40 75	16s rRNA Miseq sequencing PCR-amplification					↑ Gemmiger ↑ Parabacteroides ↑ Alistipes ↓Phascolarctobacterium ↓Ruminococcus ↑Klebsiella ↑Haemophilus ↑Gemmiger ↑Parabacteroides
Lu et al. (2011)	symptomatic carriage of hepatitis B virus (HBV) chronic hepatitis B HBV cirrhosis health controls	30 31 31 32	quantitative PCR 16S rRNA				↓Bifidobacteria ↓Lactobacillus ↓Pediococcus ↓Bifidobacteria ↓Lactobacillus ↓Pediococcus ↓Leuconostoc ↓Weissella	
Aly et al. (2016)	stage4-HCV patients healthy individuals	6 8	highthroughput 16S rRNA gene sequencing using Illumina MiSeq	↑Bacteroidetes ↑Firmicutes, ↑Proteobacteria, ↑Actinobacte ria				↑Prevotella ↑Faecalibacterium ↑Ruminococcus ↑Clostridium
Heidrich, et al. (2018)	patients infected with HCV without cirrhosis[NO CIR]; patients infected with HCV with cirrhosis [CIR]) healthy controls (HC)	57 38 50	16s rRNA gene sequencing					↑Butyricimonas ↑Victivallis ↑Veillonella ↑Lactobacillus ↑Streptococcus ↑Alloprevotella ↑Flavonifractor ↑Megasphaera ↑Acetivibrio
Inoue et al. (2018)	CHC patients healthy individuals	166 23	16S ribosomal RNA gene sequencing.			↓Clostridiales ↑Streptococcus ↑Lactobacillus.		
Ponziani et al. (2018)	patients with HCV‐related liver cirrhosis healthy controls	12 12	16s rRNA gene sequencing	↑Proteobacteria			↑Staphylococcaceae ↑Veillonellaceae ↑Enterobacteriaceae ↑Corynebacteriaceae ↑Micrococcaceae ↓Methanobacteriaceae	↑Staphylococcus ↑Dialister ↑Eubacterium ↑Enterococcus ↑Corynebacterium ↓Methanobrevibacter
Wu et al. (2022)	patients with acute hepatitis E (AHE) patients healthy controls (HCs)	33 25	high-throughput 16S ribosomal ribonucleic acid gene sequencing.	↑Proteobacteria ↑γ -Proteobacteria			↑Enterobacteriaceae	

↑:The number of a certain intestinal flora increased.

↓:The number of a certain intestinal flora decreased.

## 7 Probiotics in the treatment of hepatic diseases

Probiotics are a group of active microorganisms that have beneficial effects on the host,including: yeasts, Lactobacillus, Bifidobacterium, actinomycetes,etc., and act by modulating the host’s mucosal and systemic immune function or by regulating intestinal inflammation and flora balance.

More and more studies have found that probiotics play a unique role in the treatment of hepatitis-cirrhosis-hepatocellular carcinoma: (i)Probiotics can inhibit the translocation of endotoxin and improve endotoxemia.Through *in vivo* and *in vitro* experiments, Dai et al. (2012). found that VSL#3 probiotics increased the expression of tight junction proteins by activating p38 and ERK signaling pathways, protecting the intestinal epithelial barrier, and preventing bacterial products such as endotoxin from entering the portal circulation from the intestine. In another study of patients with HBV and HCV infection, plasma endotoxin levels were decreased by increasing the number of Bifidobacterium and Lactobacillus (Chen et al., 2007). (ii)Probiotics can reduce the toxicity of carcinogens such as aflatoxin B1 (AFB1) and zearalenone (ZEA) and reduce the risk of liver cancer.According to Huang’s research: Bacillus subtilis can degrade AFB1 and ZEA, and a higher degradation rate was observed when Bacillus subtilis, Lactobacillus casein and Candida utilis were mixed in a certain proportion (Huang et al., 2018). (iii) Probiotics can also reduce liver damage by regulating bile acids(BA).*In vivo* studies showed that Lactobacillus rhamnosus GG (LGG) can be used as a complementary treatment to increase intestinal FGF-15 expression and subsequently reduce hepatic cholesterol 7α-hydroxylase and BA synthesis in mice, and enhance urinary and faecal excretion of BA, preventing liver injury and liver fibrosis induced by excessive BA in mice. *In vitro* studies also showed that LGG inhibited the inhibitory effect of T-βMCA on the expression of FXR and FGF-19 in Caco-2 cells (Foley et al., 2021). (iv) Probiotic preparations reduce the concentration of ammonia in the arteries, improve hepatic encephalopathy (HE). Application of a probiotic preparation (Clostridium butyricum combined with Bifidobacterium infantis) to treat patients with hepatitis B-related cirrhosis combined with mild hepatic encephalopathy showed that in the probiotic treatment group, Clostridium group I and Bifidobacterium were significantly enriched, while Enterococci and Enterobacteriaceae were decreased. Indicating that probiotics promoted the growth of beneficial flora and decreased the growth of harmful flora, significant reduction in intravenous ammonia was also observed in the probiotic treatment group. In addition, the parameters of intestinal permeability (LPS and D-lactate) and Mucosal damage index (DAO) was significantly improved after probiotic treatment, which may account for the improved cognition and decreased ammonia levels (Xia et al., 2018). (v)Probiotics have beneficial effects on liver immune differentiation and carcinogenesis. Li et al. (2016), in their study establishing a mouse model of HCC, found that feeding Prohep (a novel probiotic mixture) significantly slowed tumour growth and reduced tumour size and weight by 40% compared to the control group. From a mechanistic point of view, the downregulation of IL-17 and the dramatic decrease in the level of its main producer Th17 cells play a key role in tumor suppression after probiotic feeding. Cell staining showed that the decrease in Th17 cells in the tumors of the probiotic treatment group was mainly due to the decreased frequency of migratory Th17 cells from the intestine and peripheral blood. On the other hand, this study also found that probiotics could shift the intestine microbiota toward certain beneficial flora, such as Prevotella and Oscillibacter, which could produce anti-inflammatory cytokines that inhibit Th-17 cells, reduce Th17 polarization, and promote the differentiation of anti-inflammatory Treg/Tr1 cells in the intestine. (vi) In addition, it was found that probiotics enhanced mucosal defense against pathogenic virions (Sanduzzi Zamparelli et al., 2017). Rigo-Adrover et al. (2018) established the suckling rat double- Rotavirus infection mode and found that the probiotic intervention group improved the clinical symptoms after the first infection and significantly reduced the shedding of virus. But all interventions showed a higher viral load after a second infection than the Rotavirus group, enhancing the early immune response to cope with future reinfection. Study has demonstrated that vitamin A plays an anti-norovirus role by regulating specific microbiota(Lactobacillaceae families). In an *in vitro* model of RAW264.7 cells, Lee et al. identified and demonstrated the antiviral causal effect of Lactobacillus. In a mouse model, the antiviral immune response to murine norovirus was found to be mediated by upregulation of IFN-β (Lee and Ko, 2016). In conclusion, probiotics play a positive effect in the treatment of liver inflammation and cirrhosis, and can effectively prevent the occurrence of liver cancer. It can be used as the key research direction for subsequent clinical work, with extremely broad research prospects.

## 8 Conclusions

Recent studies have shown that intestinal flora plays an important role in inducing and promoting the process of hepatitis-cirrhosis-hepatocellular carcinoma. Regulation of intestinal flora appears to be a promising therapeutic approach, but intestinal flora is a dynamic microenvironment that is affected by many factors and is difficult to be precisely regulated. More large-scale and in-depth studies are needed to fill the knowledge gap of the role of intestinal flora in liver diseases and its specific mechanism, and to translate basic theory into practical clinical application.

## Author contributions

XY was responsible for writing the outline and final review of the manuscript, and SL was responsible for writing and summarizing the entire manuscript and making the charts. All authors contributed to the article and approved the submitted version.
